# Preparation of Radiation Cross-Linked Poly(Acrylic Acid) Hydrogel Containing Metronidazole with Enhanced Antibacterial Activity

**DOI:** 10.3390/ijms21010187

**Published:** 2019-12-26

**Authors:** Jin-Oh Jeong, Jong-Soek Park, Eui Jin Kim, Sung-In Jeong, Jae Young Lee, Youn-Mook Lim

**Affiliations:** 1 Advanced Radiation Technology Institute, Korea Atomic Energy Research Institute, Jeongup-si, Jeollabuk-do 56212, Korea; jojeong86@kaeri.re.kr (J.-O.J.); sijeong@kaeri.re.kr (S.-I.J.); 2 School of Materials Science and Engineering, Gwangju Institute of Science and Technology, Gwangju 61005, Korea; jaeyounglee@gist.ac.kr; 3 Internal Medicine, Mary’s Hospital, College of Medicine, The Catholic University, Uijeongbu-si, Gyeonggi-do 11765, Korea; Jin2kim6999@daum.net

**Keywords:** drug delivery, antibacterial activity, metronidazole, poly (acrylic acid), hydrogel

## Abstract

Metronidazole (MD) is known as a periodontitis medicine and has been widely used in antibiotics for resistance to anaerobic bacteria, periodontal disease, and other threats. To treat diseases, drug delivery carriers are needed with a high bioadhesive property and enhanced drug penetration. Poly (acrylic acid) (PAA) hydrogel films have a good bioadhesive property and are able to localize the absorption site and increase the drug residence time. In this study, we fabricated a MD loaded PAA hydrogel with different MD content (0.1, 0.25, 0.5, and 1 wt%) using varying doses (25, 50, and 75 kGy) and the radiation doses (25, 50, or 75 kGy) in a one-step gamma-ray irradiation process. The chemical and physical structure were determined through a Fourier transformed infrared spectroscopy, X-ray photoelectron spectroscopy, gel content, and compressive strength. In addition, MD loaded PAA hydrogels were performed to MD release behaviors and cytotoxicity. Finally, we conducted antibacterial activity to demonstrate the prevention of growth of bacteria as a therapeutic dressing. The basic chemical structure analysis of MD was changed greatly at radiation doses of 50 and 75 kGy due to degradation by gamma-ray irradiation. However, when the absorbed dose was 25 kGy, the chemical structure analysis of MD did not change significantly, and the gel content and compressive strength of MD/PAA hydrogel were approximately 80% and 130 kPa, respectively. The MD/PAA hydrogels exhibited no cytotoxicity and good antibacterial activity against *Escherichia coli*, *Staphylococcus aureus*, and *Streptococcus mutans*. These results provide good evidence that MD/PAA hydrogel prepared by gamma-ray irradiation has potential as a competitive candidate for the therapeutic dressing.

## 1. Introduction

Metronidazole (MD) is nitroimodazole compound that has been widely used for treating infections caused by anaerobic bacteria and otitis media [1,2,3,4,5]. The nitro group of MD is activated by nitroreductases such as ferredoxin in the body. A high antibacterial property is indicated by disturbed DNA division and nucleic acid synthesis, which could be due to destruction of the helical structure of bacterial DNA [6]. Generally, the method of administering MD includes an oral administration. However, oral administration has disadvantages in that a large amount of a drug taken internally disappears during the processes of degradation and absorption [7]. Therefore, drugs are taken in more than necessary (to kill the bacteria) to keep an effective concentration in the blood, which causes various side effects.

To overcome this problem, patches (e.g., hydrogel, cellulose, and film) containing drugs have been widely developed because a patch can be applied to the skin to release the appropriate amount of drug continuously, thereby minimizing side effects [8,9]. A hydrogel is defined as a three-dimensional polymeric network that can be contained in water or body fluids [10,11]. Hydrogels have been widely used in biological applications such as wound dressings, adhesion prevention films, and contact lenses because of their marked similarity to human tissue, excellent biocompatibility, and controllable properties [12,13,14]. In addition, hydrogels maintain a stable volume in the body owing to the formation of stable structures that show little fluidity under the application of external forces. Hydrogels are fabricated using physical and chemical cross-linking processes [15,16]. Physical crosslinking is achieved by the formation of ionic bonds, hydrogen bonds, crystallization between polymer chains, or a freezing/thawing method. In the freezing/thawing method, cross-linking is achieved by the formation of crystallites in the polymer-rich domain between water and the polymer chain during repeated freeze/thaw cycles. However, the inherent disadvantage to this approach is long process time (3 or 4 days) [17]. In contrast, chemical cross-linking processes are much faster. In this case, cross-links are formed by chemical and radical reactions. Irradiation of polymer solutions results in polymer radicals, which combine through covalent bonds to form insoluble hydrogels [18].

In the case of radiation-induced cross-linking, cross links are formed at the carbon atoms of C–C bonds in the polyvinyl polymer. The radiation cross-linking method has many advantages: Cross-linking agents are not required [19,20]; no residual toxic substances are found in the hydrogel; and hydrogels having diverse properties can be fabricated by simply controlling the radiation dose, polymer concentration, and the condition of radiation exposure [21,22,23].

Hydrogels are typically composed of polymers such as natural polymers (e.g., collagen, gelatin, hyaluronic acid, or chitosan) or synthetic polymers (e.g., poly(acrylic acid) (PAA), polyethylene oxide (PEO), or poly(vinyl alcohol) (PVA) [18,19,24]. Synthetic polymers allow a higher degree of swelling and impart superior mechanical properties and adhesion strength to the hydrogels, than with natural polymers. For example, PAA hydrogels show good adhesion strength and are used in bioadhesive drug delivery systems [25,26]. PAA is a hydrophilic and highly absorbent polymer that has been widely applied in biomaterials due to its high solubility and biodegradability [15]. PAA hydrogels have been widely used as drug carriers because of their good bioadhesive properties and enhanced drug penetration. It is possible to localize the absorption site of a drug in the PAA hydrogel and to increase the drug residence time [27].

**Figure 1:**
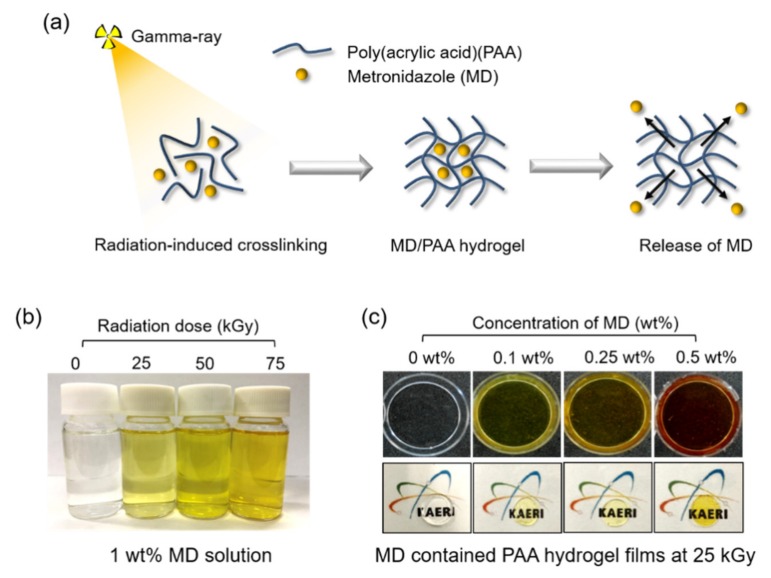
(**a**) A schematic illustration of MD/PAAc hydrogel films using gamma-ray. (**b**) Optical images of radiated 1 wt% MD solution with different radiation dose (0, 25, 50, and 75 kGy) (**c**) Optical images of MD/PAAc hydrogel film with different concentration of MD.

The aim of this paper is to develop a one-step process for PAA hydrogel with excellent biocompatibility, antibacterial activity and mechanical properties by gamma-ray as a therapeutic dressing. If the drug is loaded after preparing PAA hydrogel using gamma-ray, it requires a two-step process of hydrogel preparation and drug loading. In addition, the drug loading is limited and the drug release efficiency is difficult to expect the desired effect. In this study, the MD-containing PAA hydrogels were prepared using gamma-ray in a one-step process. The stability of the molecular structure of the drug (MD) by gamma-ray was confirmed. In addition, the MD containing PAA hydrogels were verified through chemical and physical structure analysis (such as gel content, degree of swelling, Fourier transform infrared spectroscopy (FTIR), and compressive strength); cumulative release was verified by MD analysis (Figure 1 and Table 1 and Table 2). In addition, we investigated the characteristics (such as cytocompatibility and antibacterial activity) of an MD/PAA patch as therapeutic dressing for inhibiting the proliferation of various bacteria (e.g., *Escherichia coli* (*E. coli*), *Staphylococcus aureus* (*S. aureus*), and *Streptococcus mutans* (*S. mutans*)). This study focused on the MD containing PAA hydrogel and revealed a promising approach for the development of radiation cross-linking processes.

## 2. Results and Discussion

### 2.1. Characterization of Iradiated MD

MD has four main physical characteristics: (1) it is difficult to dissolve in common solvents; (2) it has a crystalline structure, and the crystals are off-white in color; (3) it is temperature-sensitive; and (4) its color is easily changed upon exposure to light [28,29].

**Figure 2:**
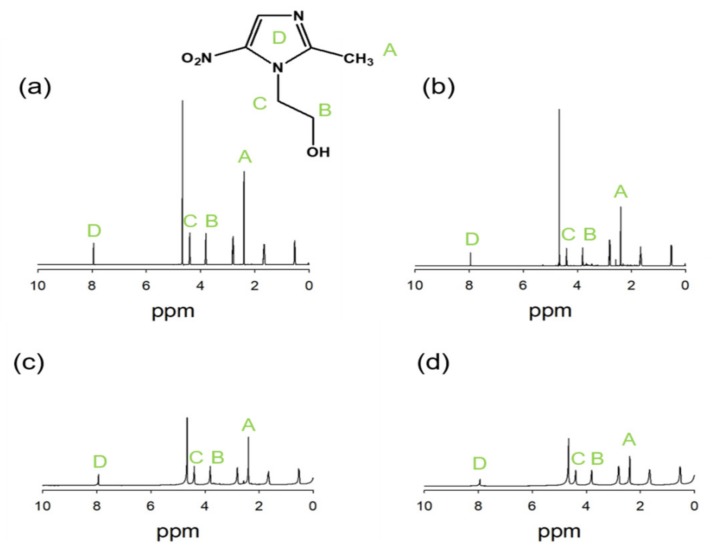
NMR spectra of MD (**a**) 0 kGy (**b**) 25 kGy, (**c**) 50 kGy, and (**d**) 75 kGy.

As shown in Figure 2, the ^1^H-NMR spectrum irradiated MD solution with various dose of 0, 25, 50, and 75 kGy were confirmed. The peak at 2.4 ppm assigned to the characteristic methyl proton. In addition, the peaks at 3.6 and 4.3 ppm were attributed to protons of CH_2_OH and NCH_2_, respectively. The peak of aromatic proton was confirmed at 8 ppm. When the radiation dose was increased, the degradation of MD was greater. However, MD irradiated at 25 kGy showed no significant change of structure to be stable to gamma-ray of dose at 25 kGy.

**Figure 3:**
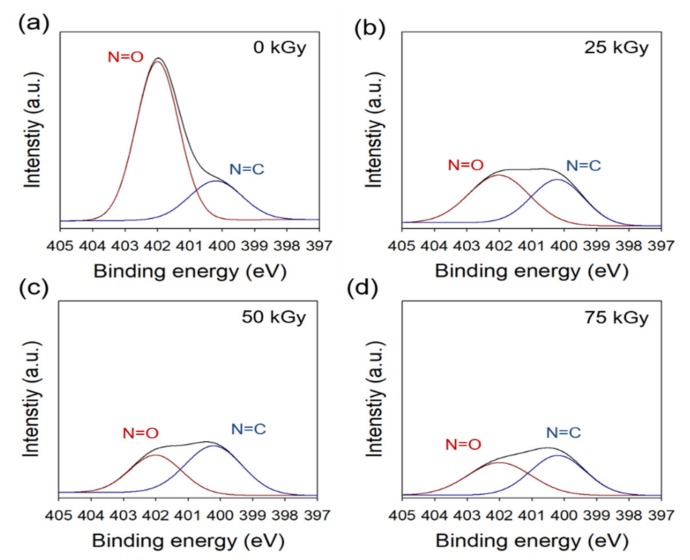
XPS spectra (N1s) of radiated metronidazole: (**a**) 0 kGy, (**b**) 25 kGy, (**c**) 50 kGy, and (**d**) 75 kGy.

Figure 3 shows the chemical composition of irradiated MD analyzed by XPS and compared with that of pristine MD. High-resolution N1s spectra of the samples were deconvoluted into two peaks; namely, 402 eV (N=O) and 400.2 eV (N=C). With an increase in radiation dose, the peak intensities at 402 eV were drastically decreased. In addition, the peak intensity was decreased with increase in the radiation dose, such as with the FTIR results. These results suggest that the MD irradiated at 25 kGy can be expected to be more stable; however, MD irradiated at more than 25 kGy is less useful as a drug due to the degradation of nitroimidazole. These results showed that N=O groups and the imidazole rings of MD were degraded by gamma rays when the radiation dose was increased, in contrast to non-radiated samples. When the radiation dose was increased, the degradation of MD was greater. Radiolysis or UV-photolysis can break down N=O groups and imidazole rings of MD [30]. In addition, when UV-photolysis was used, the degradation levels of N=O groups and imidazole rings of MD increased with increased time and light intensity, relative to the degradation by non-radiation exposed UV-photolysis [31]. On the basis of the results of UV-photolysis, it is expected that radiolysis by gamma rays would result in more degradation because the energy of gamma rays is higher than that of UV rays.

### 2.2. Characterization of MD/PAA

**Figure 4:**
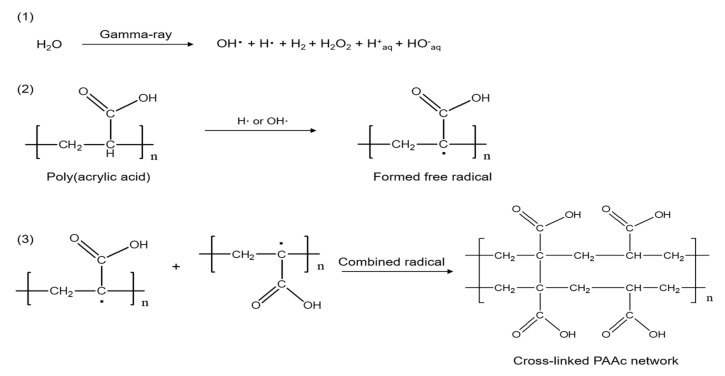
Mechanism of poly (acrylic acid) hydrogel formation using gamma-rays.

Under gamma-ray irradiation, PAA formed free radicals on the polymer chain [19]. The water molecule formed a hydroxyl radical (OH·) and a hydrogen radical (H·). The C-H bond of PAA was broken by the hydroxyl radical (OH·) to form a free radical on the PAA chain. The released hydrogen radical (H·) combined with H· from water, leading to the evolution of hydrogen gas. In addition, the free radical of PAA was able to cross-link with other PAA molecules, resulting in the formation of PAA hydrogel films [30]. The overall reaction mechanism of MD/PAA is shown in Figure 4. In addition, the color of MD/PAA hydrogel films changed to dark yellow with increase in the radiation dose from 25 to 75 kGy, due to prolonged exposure to high-energy γ-radiation. The optical images of the MD/PAA are shown in Figure 1b.

**Figure 5:**
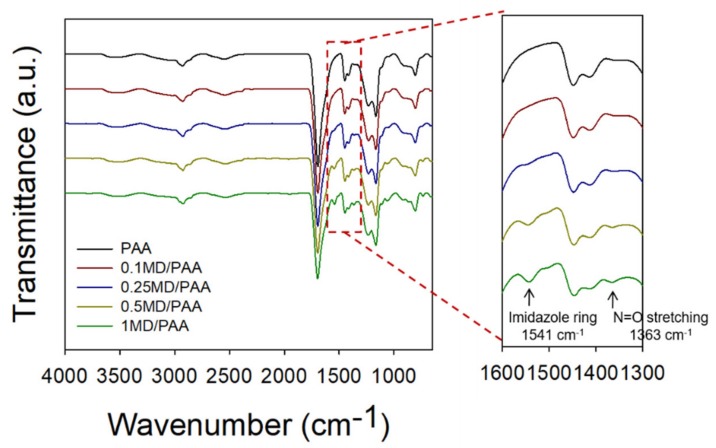
FTIR spectra of MD/PAA (metronidazole/poly (acrylic acid)) hydrogel films with different content of MD (0, 0.1, 0.25, and 0.5 wt%) and radiation dose at 25 kGy.

Chemical structure analysis of the MD/PAA samples was carried out using ATR-FTIR, and the results are shown in Figure 5. The presence of PAA was confirmed by –COOH (carboxylic acid group) and –OH (hydroxyl group) stretching vibrations at 1700 and 3400 cm^−1^, respectively [27,31]. New peaks from the nitrite group of MD/PAA were observed at 1363 and 1541 cm^−1^ due to the presence of MD [30]. The peak intensity of N=O stretching and the imidazole ring was increased with the MD concentration (from 0 to 1 wt%). This result confirms that the PAA hydrogel was successfully impregnated with MD after irradiation.

**Figure 6:**
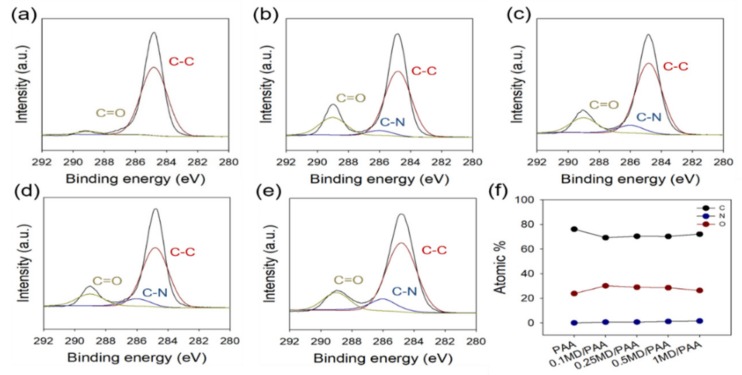
XPS spectra (C1s) of MD contained poly(acrylic acid) hydrogel films: (**a**) PAA, (**b**) 0.1MD/PAA, (**c**) 0.25MD/PAA, (**d**) 0.5MD/PAA, (**e**) 1MD/PAA (irradiated at 25 kGy), and (**f**) relative composition of carbon, nitrogen, and oxygen atoms.

Figure 6 shows that the chemical composition of MD/PAA was analyzed by XPS. High-resolution C1s spectra of the MD/PAA were deconvoluted at 284.83 eV (C-C), 286.27 eV (C-N) and 289.23 eV (C=O). A new peak at 286.27 eV (C-N) was observed and significantly increased with increase in the MD content (Figure 6b–e). While the N=O groups and imidazole rings of MD were degraded by gamma rays (based on the results of characterization of radiated MD), with increase in the MD content, active MD capable of its characteristic reaction remained in the PAA hydrogel films. This result confirms that the PAA hydrogel was successfully impregnated with MD after irradiation.

**Figure 7:**
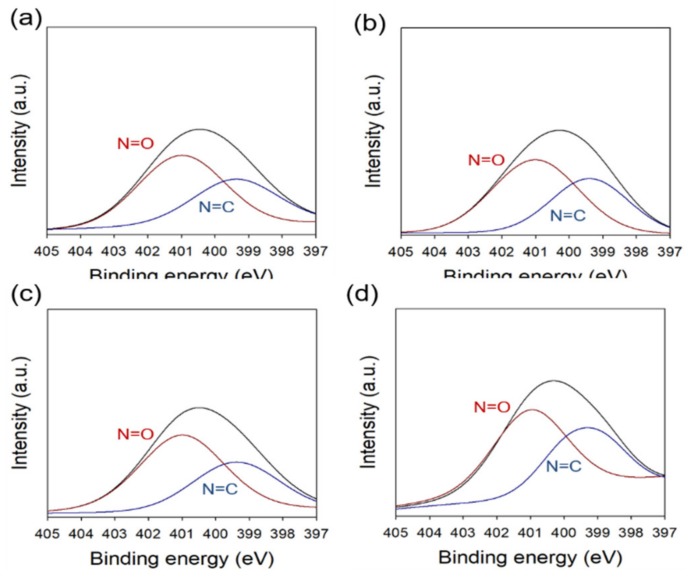
XPS spectra (N1s) of MD/PAA hydrogel film with different MD content: (**a**) 0.1 wt%, (**b**) 0.25 wt%, (**c**) 0.5 wt%, and (**d**) 1 wt%.

Figure 7 shows the chemical composition of MD/PAA hydrogel film with different MD content (0.1, 0.25, 0.5, and 1 wt%) analyzed by XPS. High-resolution N1s spectra were deconvoluted at N=O and N=C. The intensity of N=O and N=C slightly increased, and N=O was degraded at 25 kGy as the MD content increased.

**Figure 8:**
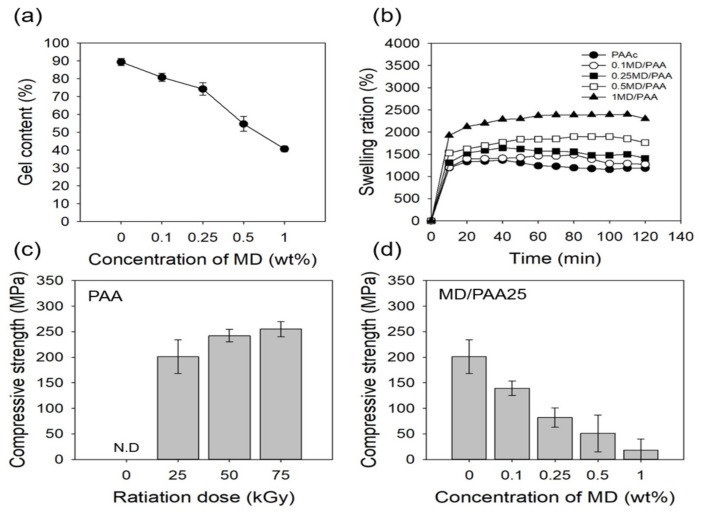
(**a**) Gel content of MD/PAA hydrogel films with different content of MD from 0 to 1 wt% with radiation dose at 25 kGy, (**b**) degree of swelling of MD/PAA hydrogel films with different content of MD from 0 to 1 wt% with radiation dose at 25 kGy, (**c**) Compressive strength of 7 wt% PAA hydrogel films with different radiation dose of 0, 25, 50, or 75 kGy, and (**d**) MD/PAA hydrogel films with different MD content (0, 0.1, 0.25, 0.5, or 1 wt%) with radiation dose at 25 kGy. (N.D = not detected).

The gel content of MD/PAA at different radiation dose and MD content is shown in Figure 8a. The gel content of PAA was as high as 98.43%, and formed a dense three-dimensional network. However, as the MD content was increased from 0.1 to 0.5 wt%, the gel content significantly decreased with irradiation at 25 kGy: the gel content for the 0.1, 0.25, and 0.5 wt% MD containing samples was 80.83%, 74.29%, 54.73%, and 40.71%, respectively. The possible reason for this result is that the crystallinity of MD interfered with the crosslinking reaction of PAA by radiation. This could have occurred because, the samples were kept at room temperature for a long period of time during the irradiation process.

Figure 8b shows that the degree of swelling of MD/PAA increased with increase in the MD content. This is because, the porous three-dimensional networks were affected by the incorporation of MD. While the degree of swelling of 1 MD/PAA was 2301%, the 0.1, 0.25 and 0.5 MD/PAA sample showed a swelling of 1275%, 1411%, and 1761%, respectively. This result suggests that the MD remained between the PAA chains, preventing crosslinking and decreasing the physical interactions between the PAA chains. However, after 60 min, the degree of swelling decreased due to destruction of the hydrogel structure by the crystalline nature of MD.

Figure 8c and d show the compressive strength of PAA and MD/PAA with different radiation dose and MD content. In this study, a 50% compression was chosen for the compressive strength. The compressive strength of PAA was found to increase with the radiation dose (204 kPa at 25 kGy), (242 kPa at 50 kGy), and (256 kPa at 75 kGy), because of the larger degree of cross-linking in the hydrogels. At 25 kGy, the compressive strength of 0.1MD/PAA was 139 kPa, while that of 1MD/PAA25 was 18 kPa, indicating a decrease of approximately 120 kPa. The same trend in compressive strength was observed at the other radiation doses. Generally, the results of compressive strength were proportional to the gel content (Figure 8a). In fact, the compressive strength of MD/PAA hydrogel was significantly decreased compared with pristine PAA hydrogel [32].

### 2.3. In Vitro Cytocompatibility of MD/PAA

**Figure 9:**
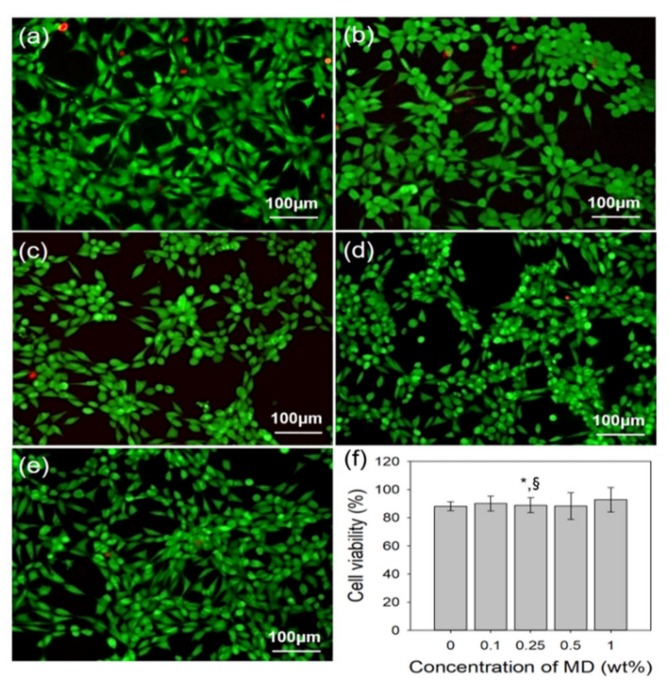
Live/Dead assay of NIH3T3 cells cultured on MD/PAA hydrogel films with different content of MD: (**a**) 0 wt%, (**b**) 0.1 wt%, (**c**) 0.25 wt%, (**d**) 0.5 wt%, and (**e**) 1 wt% irradiated at 25 kGy, and (**f**) cell viability of MD/PAA hydrogel film using WST-1 assay. (“*” indicates statistical significance relative to PAA, and “§” relative to the 0.1MD/PAA, with *p* < 0.05).

NIH3T3 fibroblast was cultured to identify the cytocompatibility of MD/PAA with increase in the MD content. When NIH3T3 was cultured for one day, the fluorescence microscopy images of MD/PAA were used to perform a live/dead assay (Figure 9a–e). The cell viability was determined using a WST-1 assay. The WST-1 reagent solution was added and incubated with 5% CO_2_at 37 °C for 3 h. The absorbance of the solution was recorded at 450 nm using a microplate reader. The survey of PAA and MD/PAA with increase in MD content confirmed that the live cells observed were >80%, indicating good cytocompatibility. There was no significant difference in the cell viability of all samples. The cell viability of 1MD/PAA was 90%. The result of cytocompatibility was showed that MD/PAA hydrogel films was no cytotoxicity and good biocompatibility due to used biocompatible polymer.

### 2.4. Release Test of MD/PAA

Drug is continuously released from polymer due to the interaction between the drug and polymer. Therefore, the compatibility of the drug and polymer is an important factor in drug release efficiency [33,34].

The surface morphologies of PAA and MD/PAA25 hydrogel films were observed by SEM, as shown in Figure S1. The hydrogel films were confirmed that were smooth morphologies regardless of contained MD or not contained MD.

**Figure 10:**
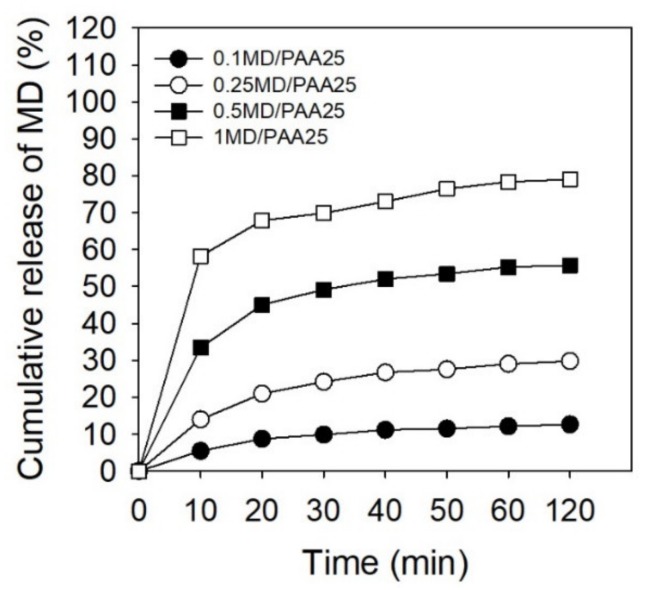
Cumulative release of metronidazole from MD/PAA hydrogel films with different content of MD (0.1, 0.25, 0.5, and 1 wt%) and radiation dose at 25 kGy.

The MD/PAA hydrogel films were dried at room temperature. The MD was released in PBS (pH 7.4) to identify cumulative release of MD with different times (10, 20, 30, 40, 50, 60, and 120 min) using UV spectroscopy and absorbance at 319 nm. The MD/PAA25 with different concentration of MD were investigated and are shown in Figure 10. The cumulative release of MD increased with increase in the MD content. Specifically, the MD was consistently released from the 1MD/PAA, reaching approximately 80% at 120 min in PBS. The value of 1MD/PAA was increased by more than approximately 65% compared to 0.1MD/PAA. However, the release of MD was comparatively high for the first 10 min after the drug began being released.

### 2.5. Anti-Bacterial Activity Test

**Figure 11:**
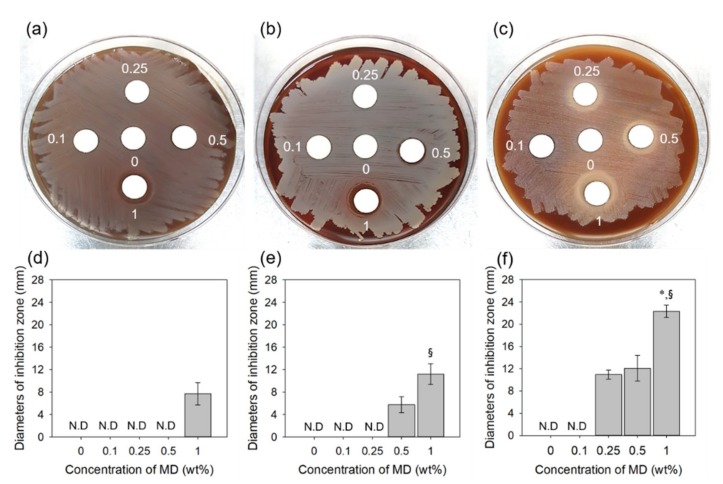
Anti-bacterial activity of the MD/PAA hydrogel films: (**a**,**d**) *Escherichia coli*, (**b**,**e**) *Staphylococcus aureus*, and (**c**,**f**) *Streptocaccus mutans*. (“*” indicates statistical significance relative to the 0.25 MD/PAA and “§” relative to the 0.5 MD/PAA, with *p* < 0.05), (N.D = Not detected).

The anti-bacterial activity of MD/PAA hydrogel films against *E. coli*, *S. aureus*, and *S. mutans* were studied using solid growth media (Figure 11). *S. mutans* is considered the cause of dental caries and periodontitis [35,36]. The MD/PAA hydrogels inhibit and kill Gram-positive *S. aureus* bacteria and Gram-negative *E. coli*. The antibacterial effects of MD/PAA hydrogel films increased with increasing MD content. The MD is easily absorbed in anaerobic organisms and reduced to polar substances of deficient nitro group by nitroreductases such as ferredoxin. This reductant shows antibacterial activity that destruct DNA helical structure by disturbance of DNA cleavage and nucleic acid synthesis [6]. In the case of *E. coli*, high anti-bacterial activity was observed with 1 MD/PAA, whereas the 0.1 MD/PAA, 0.25 MD/PAA, and 0.5 MD/PAA hydrogel films did not exhibit antimicrobial activity due to the lower MD content (Figure 11a,d). In the case of *S. aureus*, 0.5 MD/PAA and 1 MD/PAA hydrogel films showed that an inhibition zone increased with increase in MD (Figure 11b,e). In addition, the MD/PAA hydrogels showed better antimicrobial activity against *S. mutans* causing periodontitis (Figure 11c,f). PAA and 0.1MD/PAA hydrogels did not show anti-bacterial activity against *S. mutans*; however, MD/PAA with increased MD content (0.25, 0.5, and 1 wt%) exhibited observable large inhibition zones. These results indicate that a radiation dose <25 kGy does not diminish the anti-bacterial activity of MD. Finally, the above results indicate that 1MD/PAA irradiated at 25 kGy can be expected to be more stable in terms of drug release, cytocompatibility, and anti-bacterial activity, and can be utilized as an antimicrobial drug delivery system, including for treatment of periodontitis.

## 3. Materials and Methods

### 3.1. Materials

PAA (molecular weight = 100,000) was purchased from Waco Pure Chemical Industries, Ltd. (Osaka, Japan). MD was obtained from Sigma-Aldrich (St. Louis, MO, USA). All other reagents and solvents were of analytical grade and used as received.

### 3.2. Exposure of Metronidazole by Gamma-Ray

To confirm the radiation effects of MD, MD (1 wt%) was dissolved in deionized (DI) water using a shaker-incubator at 50 °C and 100 rpm, under dark conditions. Then, the MD solution was exposed to radiation using gamma ^60^Co sources (ACEL type C-1882, Korea Atomic Energy Research Institute) at different radiation doses (25, 50, and 75 kGy) at a dose rate of 10 kGy/h.

### 3.3. Preparation of Metronidazole Containing Poly (acrylic acid) Films Using Gamma-Ray

PAA was dissolved in DI water using a magnetic stirrer to a final concentration of 7 wt%. MD (0.1, 0.25, 0.5, and 1 wt%) was dissolved in the PAA solution using a shaking incubator at 50 °C at 100 rpm until perfect dissolution, under dark conditions. The MD containing PAA solution was irradiated using a ^60^Co source with different radiation doses 25, 50, and 75 kGy (10 kGy/h) to prepare the MD containing PAA hydrogel film (MD/PAA).

### 3.4. Chemical Structure

To confirm the chemical structure, the samples were measured using ATR-FTIR (Attenuated total reflection-Fourier transformed infrared spectroscopy, Bruker TEMSOR 37, Bruker AXS. INC., Karlsruhe, Germany) over the range 650 to 4000 cm^−1^.

The X-ray photoelectron spectroscopy (XPS, VG Multilab 2000 Spectrometer, Thermo Scientific, Waltham, MA, USA) spectra of the irradiated MD was confirmed compared with pristine MD. The XPS was equipped with an Al Kα X-ray and then deconvoluted N1s spectra were obtained using XPS PEAK software (University of Hong Kong, China).

The nuclear magnetic resonance (NMR) spectra of radiated MD was observed by 500 MHz ^1^H-NMR (ECA 500 MHz spectrometer, JEOL, Tokyo, Japan) with averaged over 32 scans. Then, the MD and radiated MD solution was mixed in 0.5 mL Deuterium oxide-d solution, and NMR spectra were obtained.

### 3.5. Characteristic of Hydrogel

The MD/PAA samples were dried until they were completely free of water. The MD/PAA test specimens were prepared. The initial weight of the samples was recorded, and the samples were stirred in distilled water for 24 h at room temperature (RT). Then, the gel content of the PAA and MD/PAA hydrogel was calculated from the following equation:

Gel content (%) = (W_l_/W_i_) × 100 (1)

where W_i_ and W_l_ represent the initial and final weights of the dried samples, respectively.

To measure the degree of swelling, the dried film was soaked in DI water for different time intervals at RT, until the equilibrium swelling state was reached. The degree of swelling was determined from the following equation:

Degree of swelling (%) = [(W_w_ − W_d_)/W_d_] × 100 (2)

where W_d_ and W_w_ represent the weights of the dry and wet film, respectively.

The MD/PAA sample was prepared and then compressive strength was analyzed using a texture analyzer (TA-XT2i, Stable Micro Systems, Ltd., Godalming, UK) with a load of 50 N and a working distance of 20 mm from the punch. The compressive strength was measured under 50% compression, at a crosshead speed of 5 mm/s.

Surface morphologies were investigated using scanning electron microscopy (SEM, JSM-6390, JEOL, Tokyo, Japan) with working distance of 10 mm and an electron beam of 10 kV. To observe the high resolution SEM images, samples were coated with gold for 70 s by sputter-coating.

### 3.6. In Vitro Cytocompatibility Test

An in vitro test of the extracted solution was performed according to ISO 10993-5. The extraction medium was prepared by immersing the MD/PAA in DPBS (Dulbecco’s Phosphate-Buffered Saline) at 37 °C for 24 h and filtered through a 0.22 µm to exclude free-living bacteria and archaea (Sartorius Ltd., Epsom, UK).

NIH3T3 was cultured in DMEM (Dulbecco’s Modified Eagle’s Medium) medium containing 10% FBS (Fetal Bovine Serum) and 1% AA (Antibiotic-Antimycotic) with 5% CO_2_ at 37 °C for 24 h, seeding density of 2 × 10^4^ cells/well. The culture medium was removed after 24 h and then extraction solution (diluted 2× in culture medium) was added to the 96-well plate and incubated with 5% CO_2_ at 37 °C for 24 h in humidified cell culture incubator. After cell culture, live/dead staining (LIVE/DEAD Viability/Cytotoxicity Kit, Molecular Probes Inc., Eugene, OR, USA) was performed for cytotoxicity. The culture medium was removed and washed with DPBS to stain calcein AM and ethidium homodimer-1 (diluted to 2 and 4 μm in DPBS) for incubation at 37 °C for 15 min, followed by washes with DBPS. After incubation, live/dead images were acquired using a fluorescence microscope (DMI3000B, Leica, Germany) and images were merged by Image J (NIH, MD, USA). The cell viability was determined by using a WST-1 assay. The WST-1 solution (WST-1: DMEM = 1:9) was added and incubated with 5% CO_2_ at 37 °C for 3 h. The absorbance of the solution was recorded at 450 nm using a microplate reader (Powerwave XS, Biotek, VT, USA) (*n* = 4).

### 3.7. Release Test

The MD/PAA hydrogels were dried at room temperature. Each dried sample was immersed in phosphate buffered saline (PBS) and incubated in a water bath (BS-21, JEIO TECH, Daejeon, Korea) at 37 °C for 120 min, with gentle shaking at 50 rpm. The samples were acquired at pre-determined time intervals (0, 10, 20, 30, 40, 50, 60, and 120 min), and the released MD was measured using a UV-Vis spectrophotometer (S-3100, Scinco, Seoul, Korea). The absorbance was recorded at 319 nm.

### 3.8. Anti-Bacterial Activity Test

The anti-bacterial activities of MD/PAA against *E. coli* (ATCC 43895), *S. aureus* (ATCC 14458), and *S. mutans* (ATCC 25175) were studied using a solid growth medium. A portion (100 µL) of each bacterial type was uniformly spread onto brain heart infusion broth (BHI) of sheep blood agar and further pre-incubated at 37 °C for 24 h with a density of 1 × 10^7^. To confirm the anti-bacterial activity, the MD/PAA was placed on a sterile paper disc (diameter 10 mm (Advantec Quantitative Filter Paper; Toyo Roshi Kaisha, Ltd., Tokyo, Japan) for placement on a bacteria plate. The plates were then incubated at 37 °C for 12 h, after which the images were acquired and the diameters of the inhibition zones measured by Image J (NIH, MD, USA).

### 3.9. Statistical Analysis

All data were presented as mean and standard deviation (SD) for *n* = 4. The *t*-test (Excel, Microsoft, Redmond, WA, USA) was used to assess the statistical significance of the results (*p* < 0.05).

## 4. Conclusions

In this study, PAA and MD mixtures were exposed to γ-radiation. The presence of MD in PAA hydrogels were verified by chemical and physical structure analysis, and cumulative release of MD was determined by MD analysis. The basic chemical structure analysis of MD was changed greatly at radiation doses of 50 and 75 kGy due to degradation by gamma-ray irradiation. However, when the absorbed dose was 25 kGy, the chemical structure analysis of MD did not change significantly. When the absorbed dose was 25 kGy, the MD/PAA hydrogel showed sufficient properties such as gel content and strength to be used in dressing materials. The MD from the PAA hydrogel was consistently released and reached to approximately 80% at 120 min. The release of MD was comparatively high for the first 10 min after drug-release began. The MD/PAA hydrogels exhibited no cytotoxicity and good antibacterial activity against *E. coli*, *S. aureus*, and *S. mutans*. These results provide good evidence that MD/PAA hydrogel prepared by gamma-ray irradiation has potential as a competitive candidate for therapeutic dressings.

## Figures and Tables

**Table 1:** Characteristics of metronidazole containing poly (acrylic acid) hydrogel films with different radiation doses.

Sample	MD Content (wt%)	Radiation Dose (kGy)
MD/PAA25	1	25
MD/PAA50	1	50
MD/PAA75	1	75

**Table 2:** Characteristics of metronidazole containing poly (acrylic acid) hydrogel films with different MD content.

Sample	MD Content (wt%)	Radiation Dose (kGy)
0.1MD/PAA	0.1	25
0.25MD/PAA	0.25	25
0.5MD/PAA	0.5	25
1MD/PAA	1	25

## Supplementary Materials

Supplementary materials can be found at https://www.mdpi.com/1422-0067/21/1/187/s1.
