# Supplementary material for: Preparation of Radiation Cross-Linked Poly(Acrylic Acid) Hydrogel Containing Metronidazole with Enhanced Antibacterial Activity
Source: Int J Mol Sci. 2019 Dec 26;21(1):187. doi: 10.3390/ijms21010187 (PMC6981512; doi:10.3390/ijms21010187)
Supplement: Supplementary file 1 [file ijms-21-00187-s001.pdf]

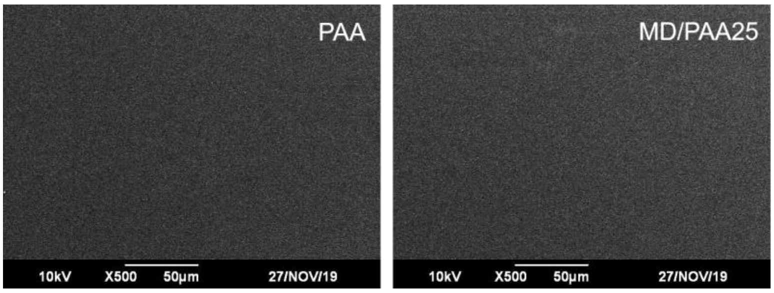

Figure S1. SEM images of PAA and 1 wt% MD containing PAA hydrogel films at radiation dose of 25 kGy.

Methods

Surface morphologies were investigated using scanning electron microscopy (SEM, JSM-6390, JEOL, Japan) with working distance of 10 mm and an electron beam of 10 kV. To observe the high resolution SEM images, samples were coated with gold for 70 s by sputter-coating.

Results

The drug-containing hydrogel films can be applied as a therapeutic dressing to release the drug when attached to a particular target and the film represents smooth morphologies. The surface morphologies of PAA and MD/PAA25 hydrogel films were observed by SEM, as shown in Figure S1. The hydrogel films were confirmed that were smooth morphologies regardless of contained MD or not contained MD.
